# Prevalence of human cytomegalovirus, polyomaviruses, and oncogenic viruses in glioblastoma among Japanese subjects

**DOI:** 10.1186/1750-9378-10-3

**Published:** 2015-01-27

**Authors:** Yumiko Hashida, Ayuko Taniguchi, Toshio Yawata, Sena Hosokawa, Masanao Murakami, Makoto Hiroi, Tetsuya Ueba, Masanori Daibata

**Affiliations:** Department of Microbiology and Infection, Kochi Medical School, Kochi University, Nankoku, Kochi, 783-8505 Japan; Division of Hematology and Respiratory Medicine, Kochi Medical School, Kochi University, Nankoku, Kochi, 783-8505 Japan; Department of Neurosurgery, Kochi Medical School, Kochi University, Nankoku, Kochi, 783-8505 Japan; Laboratory of Diagnostic Pathology, Kochi Medical School, Kochi University, Nankoku, Kochi, 783-8505 Japan

**Keywords:** Oncogenic viruses, HCMV, HPV, Polyomaviruses, Glioblastoma, Epidemiology

## Abstract

**Background:**

The association between human cytomegalovirus (HCMV) and glioblastoma multiforme (GBM) is becoming a new concept. However, information on the geographic variability of HCMV prevalence in GBM remains scarce. Moreover, the potential roles of various viruses, such as polyomaviruses and oncogenic viruses, in gliomagenesis remain unclear. Our aim was to investigate the prevalence of HCMV in GBM among Japanese patients. Furthermore, this was the first study that evaluated infection with four new human polyomaviruses in GBMs. This study also provided the first data on the detection of human papillomavirus (HPV) in GBM in the Eastern world.

**Methods:**

We measured the number of various viral genomes in GBM samples from 39 Japanese patients using real-time quantitative PCR. The tested viruses included HCMV, Merkel cell polyomavirus, human polyomavirus (HPyV) 6, HPyV7, HPyV9, Epstein–Barr virus, human herpesvirus 8, and HPV. Our quantitative PCR analysis led to the detection of eight copies of the HCMV DNA mixed with DNA extracted from 10^4^ HCMV-negative cells. The presence of HCMV and HPV genomes was also assessed by nested PCR. Immunohistochemical study was also carried out to detect HPV-derived protein in GBM tissues.

**Results:**

The viral DNAs were not detectable, with the exception of HPV, which was present in eight out of 39 (21%) GBMs. All HPV-positive cases harbored high-risk-type HPV (HPV16 and HPV18). Moreover, the HPV major capsid protein was detected in GBM tumor cells.

**Conclusions:**

In contrast with previous reports from Caucasian patients, we did not obtain direct evidence in support of the association between HCMV and GBM. However, high-risk-type HPV infection may play a potential etiological role in gliomagenesis in a subset of patients. These findings should prompt further worldwide epidemiological studies aimed at defining the pathogenicity of virus-associated GBM.

## Background

A significant portion of human cancer cases worldwide, an estimated 15%–20%, may be attributed to viral infection [1]. Several studies have indicated an exciting connection between human cytomegalovirus (HCMV) infection and glioblastoma multiforme (GBM), which is the most malignant primary brain tumor of human adults. In these studies, HCMV gene sequences were detected in >90%–95% of cases of GBM, and lower levels of HCMV expression in tumors were associated with longer survival of patients with GBM [2–8]. Furthermore, recent studies demonstrated a survival benefit in patients with GBM who received standard therapy for GBM in combination with antiviral valganciclovir treatment [9, 10], although this remains to be confirmed by additional studies. Thus, the role of HCMV in the pathogenicity of GBM is attracting increasing interest.

In contrast with these findings, several conflicting reports indicated a lack of association between HCMV and GBM [11–13]. A more recent study used next-generation sequencing to show that HCMV sequences are absent in high-grade gliomas [14]. Thus, the association between HCMV and GBM reported by some but not all studies highlights the need for additional worldwide surveys. In fact, to date, all investigations of links between HCMV and GBM have stemmed from American and European populations, and the prevalence of HCMV in GBMs in other regions of the world, such as Asia, has not been clarified. Recently, Yamashita *et al*. [15] collected GBM samples from hospitals located in the Nagoya area of Japan and tested them for the presence of the HCMV genome; however, none of their cases had detectable HCMV DNA sequences. To determine the frequency at which HCMV is present in Asian populations, we further investigated the prevalence of HCMV and viral load in GBMs from Japanese subjects, using highly sensitive quantitative PCR.

If HCMV infection is an important risk factor for the development of most cases of GBM, it would be worth exploring whether other infectious pathogens coexist with HCMV in these tumors, possibly as cofactors of pathogenesis. For example, several studies suggested that some polyomaviruses, such as the simian virus 40 (SV40) and JC virus (JCV), are associated with GBM [16, 17]. In this context, we explored for the first time the existence of newly discovered human polyomaviruses, including Merkel cell polyomavirus (MCPyV), human polyomavirus 6 (HPyV6), HPyV7, and HPyV9, in GBM samples. MCPyV is the first oncogenic polyomavirus and was originally found in Merkel cell carcinoma [18]; subsequent studies showed that MCPyV DNA was present in other malignancies, such as cervical cancer and lung cancer [19, 20]. In addition, we defined the prevalence of three human oncogenic viruses in GBMs: Epstein–Barr virus (EBV); human herpesvirus 8 (HHV8); and human papillomavirus (HPV). Our study provided the first data on the detection of high-risk-type HPVs in GBMs in the Eastern world.

## Results

### Quantitative PCR for HCMV in GBMs

To detect HCMV DNA, we employed quantitative PCR targeting two different HCMV regions using the primers and probes for major immediate early (IE) and glycoprotein B (gB) genes [21, 22]. The primers and probes were verified using DNA from a plasmid containing the HCMV IE or gB genes, DNA extracted from the HCMV AD169 strain (Advanced Biotechnologies, Columbia, MD, USA), and DNA from the AD169 strain admixed with cellular DNA. A standard curve for HCMV was generated using serial dilutions of the genome of the AD169 strain. Our quantitative PCR analysis detected at least 1.5 HCMV copies per PCR reaction (i.e., 0.0008 copies per cell) from the AD169 strain DNA admixed with 200 ng of cellular DNA extracted from HCMV-negative HEK293 cells. The number of cells in each sample was assessed by comparing the amounts of RNaseP product. The estimated number of cells per PCR reaction in the GBM samples varied from 5 to 7315 (median, 301; mean, 1028) cells. For positive controls of HCMV detection, three formalin-fixed paraffin-embedded (FFPE) samples were obtained from patients with HCMV pancreatitis (denoted as HCMV-P1), HCMV enteritis (HCMV-P2), and HCMV pneumonia (HCMV-P3), respectively. The number of HCMV copies in these positive controls was 415, 472, and 3852 copies per reaction, or 24.1, 3.82, and 399.55 copies per cell, respectively. In clear contrast, the HCMV IE and gB genes were not detectable in any of the 39 GBMs. All samples were tested at least in triplicate. The results are summarized in Table 1.

**Table 1:** **Results of real-time quantitative PCR to detect various viruses in the 39 patients with GBM and in positive control samples**

			Viral DNA copy number*												
Case no.	Age	Sex	HCMV			MCPyV	HPyV6	HPyV7	HPyV9	EBV	HHV8	HPV			Cell number
			gB	IE	PCR**							Type 16	Type 18	PCR***	
1	60	F	0	0	ND	0	0	0	0	0	0	0	0	ND	32
2	58	F	0	0	ND	0	0	0	0	0	0	0	0	ND	676
3	51	M	0	0	ND	0	0	0	0	0	0	0	0	type 16	417
4	74	F	0	0	ND	0	0	0	0	0	0	0	0	ND	1280
5	72	M	0	0	ND	0	0	0	0	0	0	0.13	0	type 16	21
6	68	F	0	0	ND	0	0	0	0	0	0	0	0	ND	135
7	49	M	0	0	ND	0	0	0	0	0	0	0	0	ND	770
8	71	M	0	0	ND	0	0	0	0	0	0	0	0	type 16	118
9	60	M	0	0	ND	0	0	0	0	0	0	0	0	ND	370
10	42	M	0	0	ND	0	0	0	0	0	0	0	0	ND	34
11	42	M	0	0	ND	0	0	0	0	0	0	0	0	ND	221
12	82	F	0	0	ND	0	0	0	0	0	0	0	0	ND	1236
13	40	M	0	0	ND	0	0	0	0	0	0	0	0	ND	48
14	77	M	0	0	ND	0	0	0	0	0	0	0	0	ND	301
15	62	F	0	0	ND	0	0	0	0	0	0	0	0	ND	454
16	75	M	0	0	ND	0	0	0	0	0	0	0	0	ND	138
17	73	M	0	0	ND	0	0	0	0	0	0	0	0	ND	26
18	43	F	0	0	ND	0	0	0	0	0	0	0	0	ND	157
19	50	F	0	0	ND	0	0	0	0	0	0	0	0	ND	133
20	46	M	0	0	ND	0	0	0	0	0	0	0	0	ND	5
21	82	M	0	0	ND	0	0	0	0	0	0	0	0	ND	114
22	4	F	0	0	ND	0	0	0	0	0	0	0	0	ND	954
23	46	M	0	0	ND	0	0	0	0	0	0	0	0	ND	533
24	70	F	0	0	ND	0	0	0	0	0	0	0	0	ND	590
25	65	F	0	0	ND	0	0	0	0	0	0	0	0	ND	33
26	59	F	0	0	ND	0	0	0	0	0	0	0	0	type 16	400
27	72	M	0	0	ND	0	0	0	0	0	0	0	0	ND	147
28	77	M	0	0	ND	0	0	0	0	0	0	0	0	ND	272
29	65	F	0	0	ND	0	0	0	0	0	0	0	0.05	type 18	50
30	72	M	0	0	ND	0	0	0	0	0	0	0	0	ND	214
31	77	M	0	0	ND	0	0	0	0	0	0	0	0	type 16	388
32	51	F	0	0	ND	0	0	0	0	0	0	0	0	ND	2637
33	77	F	0	0	ND	0	0	0	0	0	0	0	0	ND	5269
34	80	M	0	0	ND	0	0	0	0	0	0	0	0	ND	7022
35	81	M	0	0	ND	0	0	0	0	0	0	0	0	ND	3510
36	63	F	0	0	ND	0	0	0	0	0	0	0	0	ND	3205
37	70	M	0	0	ND	0	0	0	0	0	0	0	0	type 16	7315
38	70	M	0	0	ND	0	0	0	0	0	0	0	0	ND	705
39	88	F	0	0	ND	0	0	0	0	0	0	0	0.18	type 18	170
HCMV-P1^a^			827.37	2.01											17
HCMV-P2			671.98	270.96											123
HCMV-P3			3955.12	3748.20											10
MCPyV-P1						25879.50									188
MCPyV-P2						28.95									275
HPyV6-P1							107.60								92
HPyV6-P2							2.12								10
HPyV6-P3							0.20								254
HPyV7-P1								0.12							26
HPyV7-P2								0.45							10
HPyV7-P3								0.45							672
EBV-P1										61315.24					2316
EBV-P2										47466.54					2520
HHV8-P1											49621.12				1624
HPV16-P1												3.66			32
HPV16-P2												235.94			228
HPV16-P3												491.46			348
HPV16-P4												46.06			522
HPV18-P1													0.11		496
HPV18-P2													82.98		572
HPV18-P3													4.58		1191
HPV18-P4													0.04		10

*Viral DNA copy number is shown as per reaction.

**Results of nested PCR are also shown.

***Results of nested PCR and the HPV type are shown.

^a^Positive controls are as follows: HCMV-P1, HCMV^+^ pancreatitis (FFPE); HCMV-P2, HCMV^+^ enteritis (FFPE); HCMV-P3, HCMV^+^ pneumonia; MCPyV-P1, MCPyV^+^ Merkel cell carcinoma (FFPE); MCPyV-P2, MCPyV^+^ cutaneous squamous cell carcinoma (FFPE); HPyV6-P1, HPyV6^+^ basal cell carcinoma (FFPE); HPyV6-P2, HPyV6^+^ melanoma (FFPE); HPyV6-P3, HPyV6^+^ cutaneous squamous cell carcinoma (FFPE); HPyV7-P1, HPyV7^+^ basal cell carcinoma (FFPE); HPyV7-P2, HPyV7^+^ melanoma (FFPE); HPyV7-P3, HPyV7^+^ cutaneous squamous cell carcinoma (FFPE); EBV-P1 and -P2, EBV^+^ lymphoblastoid cells (cell lines); HHV8-P1, HHV8^+^ KS-1 (cell line); HPV16-P1 and -P2, HPV16^+^ cervical cancer (FFPE); HPV16-P3 and –P4, HPV16^+^ oropharyngeal cancer (FFPE); HPV18-P1 and –P2, HPV18^+^ cervical cancer (FFPE); HPV18-P3 and –P4, HPV18^+^ oropharyngeal cancer (FFPE).

F: female; M: male; ND: not detected.

We also analyzed the GBM samples for the existence of HCMV DNA by nested PCR with primer sets targeting the HCMV gB gene, as previously reported [2, 23]. Likewise, HCMV DNA was not detectable in any of our GBM samples (Table 1).

### Detection of human polyomaviruses and oncogenic viruses

Next, we tested the GBM samples for the presence of four human polyomaviruses (MCPyV, HPyV6, HPyV7, and HPyV9) and three oncogenic viruses (EBV, HHV8, and HPV). These viral DNAs were not detected, with the exception of HPV, which was detected in three out of 39 GBMs by real-time quantitative PCR targeting HPV16 and HPV18 sequences (Table 1). One patient was infected with HPV16 and two patients with HPV18, and the viral copy numbers ranged from 0.05 to 0.18 per PCR reaction, or from 0.001 to 0.006 per cell. For positive controls of HPV detection, eight FFPE samples were obtained from the following patients: four cervical cancers (two patients with HPV16 and two with HPV18 infection) and four oropharyngeal cancers (two patients with HPV16 and two with HPV18 infection). The HPV copy number of these positive control tissues ranged from 0.04 to 491.46 copies per reaction, or from 0.0002 to 1.41 copies per cell (Table 1). Thus, the HPV copy number in HPV-positive GBMs tended to be lower than those observed in the HPV-infected cervical cancers and oropharynx cancers that were used as positive controls.

Cases of GBM may be infected with HPVs other than HPV16 and HPV18 [24]. Therefore, to determine the detection frequency and types of HPV, the GBM samples were also subjected to standard PCR using the primers that were employed for the quantitative PCR assay as well as GP5+/GP6+ primers [25], or to nested PCR using the first primers MY09/11 and the second primers GP5+/GP6+ [25, 26]. The PCR products were sequenced directly, and the sequencing data obtained were BLAST searched using the National Center for Biotechnology Information (NCBI) database. Overall, eight out of 39 (21%) samples were positive for HPV by nested PCR, and all positive cases carried high-risk-type HPV. Six patients were infected with HPV16 and two patients with HPV18.

To explore whether contamination of the normal brain tissues affected the PCR results, we examined normal brain tissues from two GBM cases that harbored the HPV genome (cases 29 and 37). The normal brain tissues were obtained during surgery and stocked as FFPE tissues. As a result, HPV DNA was not detected in the non-neoplastic tissues, suggesting that that the HPV positivity resulted from HPV-infected GBM tumor cells.

**Figure 1:**
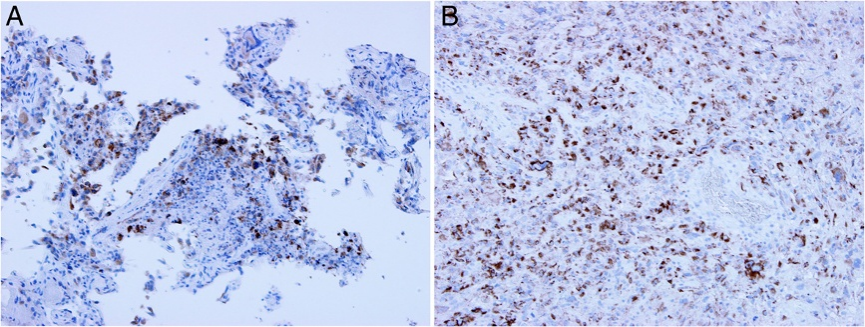
**Immunohistochemical staining of GBM tumor cells for HPV major capsid protein.** Immunohistochemical analysis using the K1H8 monoclonal antibody confirmed the expression of the HPV major capsid protein in the nucleus of tumor cells, partially in the cytoplasm. **(A)** Case 29; **(B)** Case 37. Original magnification × 100.

To evaluate the expression of HPV antigen, immunohistochemistry was performed using the mouse monoclonal antibody K1H8 against the HPV major capsid protein [27, 28]. This antibody is a broadly-reactive antibody to the following HPV types: 6, 11, 16, 18, 31, 33, 42, 51, 52, 56, and 58. The immunohistochemical study was carried out on three GBM samples, cases 29, 37, and 39, for which enough material was available. These cases were shown to be positive for HPV16 or HPV18 DNA by PCR assays (Table 1). Sample of HPV-positive cervical cancer (HPV16-P2) was used as a positive control. All cases showed positivity for staining of the HPV major capsid protein (Figure 1), indicating that the GBM tumor cells expressed the HPV-derived antigen. The immunochemical signal was detected in the nucleus of tumor cells, partially in the cytoplasm.

## Discussion

The presence of HCMV in GBM was first reported by Cobbs and colleagues [2]. Since this pioneer report, a controversy was generated regarding the presence or absence of this virus in this type of tumor [11–13]. Further studies argued that the discrepancy of the results was possibly related to technical reasons, such as differences in the sensitivity of the methods used by different groups, and a consensus was reached that HCMV sequences exist in most, but not all, GBMs [29]. However, this consensus was achieved based on findings from Caucasian patients with GBM, and information on the geographic variability of HCMV prevalence in this type of tumor is scarce. In this study, we measured the HCMV loads in both FFPE and frozen GBM tumors from 39 Japanese patients using highly sensitive quantitative PCR. Our quantitative PCR analysis detected eight copies of HCMV DNA mixed with DNA extracted from 10^4^ HCMV-negative cells. No cases harbored detectable HCMV genomes. Furthermore, nested PCR assays did not detect HCMV in GBM tissues. These results indicate that HCMV is unlikely to play a direct role in the pathogenesis of GBM in cases collected in the south of Japan. Recently, another research group was also unable to demonstrate the presence of HCMV genomes in GBM samples from a population of patients from the central region of Japan [15]. The absence of HCMV in Japanese patients with GBM shown by these two independent studies raises the possibility that the prevalence of HCMV in GBM varies among populations with different ethnic origins. Worldwide epidemiology studies have shown that individuals of European descent are more frequently affected by GBM than are those of Asian or African descent [30, 31]. In the US, the incidence of GBM is two or more times greater in white than in black people [30, 32]. Thus, differences in incidence between Western and Asian countries and among ethnic groups residing in the US suggest a genetic predisposition to GBM. GBM is a tumor that arises from glial cells. If HCMV, which is a ubiquitous herpesvirus that is present in the majority of the population worldwide, is a predominant risk factor for GBM, it is conceivable that the susceptibility of glial cells to HCMV infection might vary according to race. It is known that clinical HCMV isolates display genetic polymorphisms in multiple genes, which are supposed to be related to strain-specific tissue tropism and to the ability to established persistent infection [33]. The most polymorphic genes are those that encode viral envelope glycoproteins, which play important roles in virus entry and in cell-to-cell virus spread [33, 34]. A survey showed that the genotypic variants of the gene that encodes glycoprotein N can be divided into four groups according to the geographical provenance of clinical HCMV isolates, i.e., European, Northern American, Australian, and Chinese types [35]. Thus, genetic polymorphisms in variants isolated from geographically and migrationally different regions might be implicated in different HCMV-related pathogeneses according to race. Therefore, our present findings should prompt further investigation of the prevalence of HCMV in a series of GBM samples from diverse regions of the world, especially in Asia and Africa, as well as studies aimed at determining whether the different detection rates of HCMV are caused by ethnic variation, rather than technical sensitivity.

To date, three polyomaviruses, SV40, JCV, and BKV, have been suggested to be associated with the development of brain tumors [1, 36, 37]. A recent serological study showed a significant association between SV40 and a subset of GBM cases [16]. Because serological cross-reactivity is known among the polyomavirus family [38], it is worthwhile to test the association between new human polyomaviruses and GBM. Furthermore, it has been proposed that polyomaviruses play a synergistic role with other viruses in oncogenic transformation [39]. In this context, it is worth investigating whether HCMV-infected GBM is also coinfected with human polyomaviruses. In this study, we explored for the first time the existence of four newly discovered human polyomaviruses, MCPyV, HPyV6, HPyV7, and HPyV9, in GBMs. However, the polyomavirus DNA sequences were not detectable, suggesting that these polyomaviruses are unlikely to play a pathogenetic role in our patients with GBM.

We also searched for the viral DNAs of two oncogenic herpesviruses, EBV and HHV8, in GBMs, with negative results. Recently, Cimino *et al*. [14] reported that the EBV DNA sequence was found in five out of 21 (24%) patients from the US with high-grade gliomas using next-generation sequencing analysis. Although the discrepancy in the EBV prevalence rates might be explained in part by geographic epidemiological variations in patients, or merely by differences in the technical approaches used, our study suggests that EBV and HHV8 are not key pathogens in the development of GBM.

HPV is a common oncogenic virus in human. Recently, Vidone *et al.* [24] reported that HPV DNA was detected in 12 out of 52 Italian patients with GBM (23%) using nested PCR. Among their 12 HPV-positive cases, three cases were infected with HPV16 and nine were infected with HPV6 (low-risk type). In the present study, we found eight HPV-positive GBMs (21%). The detection rate was very similar to that obtained in the Italian population. In contrast, our study showed that the detectable HPVs were all of high-risk types (HPV16 and HPV18). Importantly, we measured for the first time the HPV viral loads in GBM tissues, and found that the copy numbers of infected HPV were relatively low compared with those detected in the HPV-positive cervical cancers and oropharyngeal cancers that were used as positive controls. The HPV genome was not detectable in controlled normal brain tissues. Currently, it is unknown whether persistent HPV infection is directly associated with gliomagenesis in a subset of patients. However, detection of the HPV major capsid protein suggest that production of viral protein from HPV genome is ongoing in GBM tumor cells. Otherwise, HPV might be just a transient ‘hit and run’ infectious pathogen, which would explain the small number of HPV-positive GBMs and lower viral loads detected in the tumor tissues. According to this theory, HCMV may first begin to proliferate in the glial cells and then it may cause cell proliferation. Additional cellular gene mutations during tumor progression may in part render HCMV expression dispensable for gliomagenesis.

## Conclusions

This was the first presentation of data regarding the prevalence of various viruses, including HCMV, human oncogenic viruses (EBV, HHV8, HPV, and MCPyV), and new human polyomaviruses (HPyV6, HPyV7, and HPyV9) in GBMs from an Asian population. In contrast with many previous reports based on Caucasian patients, we were not able to obtain direct evidence to support the association between HCMV and GBM. Furthermore, it is unlikely that GBM is associated with human oncogenic viruses, including EBV, HHV8, and MCPyV. However, our investigations demonstrated the presence of the viral genome and protein of high-risk-type HPVs in a subset of patients with GBMs. These findings should stimulate further large-scale worldwide epidemiological and virological studies aimed at defining the pathogenicity of HPV-positive GBM.

## Methods

### Patients and samples

This study included 39 Japanese patients with GBM grade IV (22 men and 17 women). The median age of the patients was 68 years (range, 4–88 years). Thirty-one FFPE tumor tissues (denoted as cases 1–31) and eight frozen tumor tissues (cases 32–39) were obtained from patients from the Kochi University Hospital. The patients resided in the Kochi prefecture, which is located on Shikoku Island in southern Japan. This study was approved by the Ethics Committee of Kochi Medical School, Kochi University.

### DNA extraction and detection of viral DNAs

For DNA extraction from FFPE tumors, three 5-μm-thick slices were prepared for DNA extraction using a ReliaPrep FFPE gDNA Miniprep System (Promega, Tokyo, Japan). From frozen tumor samples or cultured cells, DNA was isolated using a QIAamp DNA Mini Kit (Qiagen, Tokyo, Japan).

Two hundred nanograms of extracted DNA was analyzed for the detection and quantification of eight different viruses (HCMV, MCPyV, HPyV6, HPyV7, HPyV9, EBV, HHV8, and HPV) via TaqMan-based real-time PCR using a StepOne Plus Real-Time PCR System (Life Technologies, Tokyo, Japan). Real-time PCR reactions and quantitative analyses were performed primarily based on methods described previously [21, 22, 40–45]. The primers and probes used in this study are shown in Table 2. The reaction mixture used for all real-time PCR assays was prepared as follows: TaqMan Gene Expression Master Mix (Life Technologies), 900 nM of each primer, 250 nM dual-labeled probe, and 200 ng of DNA. The PCR conditions were as follows: 50°C for 2 min and 95°C for 10 min, followed by 50 cycles of 95°C for 15 s and 60°C for 1 min. Collected data were analyzed using StepOne Software v2.2 (Life Technologies). The housekeeping gene *RNaseP* was used as an internal control, and the PCR mixture without the template DNA was used as a negative control for each experiment.

**Table 2:** **Sequences of the primers and probes used for real-time quantitative PCR analysis**

Target		Sequence (5′ → 3′)		Reference
HCMV	gB	F	GGCGAGGACAACGAAATCC	[21]
		R	TGAGGCTGGGAAGCTGACAT	
		probe	FAM-TTGGGCAACCACCGCACTGAGG-TAMRA	
	IE	F	GACTAGTGTGATGCTGGCCAAG	[22]
		R	GCTACAATAGCCTCTTCCTCATCTG	
		probe	FAM-AGCCTGAGGTTATCAGTGTAATGAAGCGCC-TAMRA	
MCPyV	ST	F	GCAAAAAAACTGTCTGACGTGG	[40]
		R	CCACCAGTCAAAACTTTCCCA	
		probe	FAM-TATCAGTGCTTTATTCTTTGGTTTGGATTTC-TAMRA	
HPyV6	LT	F	TGGTCCCCTTTTGTAACAGC	[41]
		R	GCCAGAATTGCCAGAGGATA	
		probe	FAM-TGCAAACATGGCTTATGCAGAAA-TAMRA	
HPyV7	LT	F	ACTGGTTCCCACCAAATGAG	[41]
		R	TGCATAAACCAGGCCTTAAAA	
		probe	FAM-CACCCTTTTTGCAAAAGCCTTT-TAMRA	
HPyV9	VP1	F	TGCTGTTGATATTGTTGGAATTCA	[42]
		R	AACAACCCGTTTCCTTAGAGTTACA	
		probe	FAM-CTGGAGAGGCCTACCT-NFQ-MGB	
EBV	LMP1	F	GTTGATCTCCTTTGGCTCCTC	[43]
		R	GTGTCTGCCCTCGTTGG	
		probe	FAM-TTGTTGAGGGTGCGGGAGGGAGTCATCGTGG-TAMRA	
HPV16	E6	F	AGGACCCACAGGAGCGAC	[44]
		R	AGTCATATACCTCACGTCGCAGT	
		probe	FAM-ATGCACAGAGCTGCAAACAA-TAMRA	
HPV18	L1	F	GGTTCAGGCTGGATTGCG	[44]
		R	TACACGCACACGCTTGGC	
		probe	FAM-TCGCAAACGTTCTGCTCC-TAMRA	
HHV8	ORF26	F	AGCCGAAAGGATTCCACCAT	[45]
		R	TCCGTGTTGTCTACGTCCAG	
		probe	FAM-TGCAGCAGCTGTTGGTGTACCACAT-TAMRA	
RNase P		F	AGATTTGGACCTGCGAGCG	[40]
		R	GAGCGGCTGTCTCCACAAGT	
		probe	FAM-TTCTGACCTGAAGGCTCTGCGCG-TAMRA	

### DNA sequencing analysis

After the amplification of DNA by standard PCR or nested PCR, the PCR products were purified with a High Pure PCR Product Purification Kit (Roche Diagnostics, Tokyo, Japan) and then sequenced directly using an ABI Prism BigDye Terminator v1.1 Cycle Sequencing Kit (Life Technologies). The sequenced products were analyzed using a model 3130 Genetic Analyzer (Life Technologies). The nucleotide sequences obtained were aligned and edited with BioEdit software (Ibis Biosciences, Carlsbad, CA, USA).

### Immunohistochemistry

To detect HPV protein, immunohistochemistry was performed on FFPE tissue sections using a mouse monoclonal antibody, K1H8 against the HPV major capsid protein [27, 28]. The immunohistochemical study was carried out using a VENTANA DISCOVERY autostainer system according to the protocol provided by the manufacturer (Roche Diagnostics, Tokyo, Japan). The antomated protocol is based on an indirect biotin-avidin system using a biotinylated universal secondary antibody and diaminobenzidine substrate with hematoxylin counterstainig.

## Abbreviations

HCMV: Human cytomegalovirus
GBM: Glioblastoma multiforme
SV40: Simian virus 40
JCV: JC virus
MCPyV: Merkel cell polyomavirus
HPyV6: Human polyomavirus 6
HPyV7: Human polyomavirus 7
HPyV9: Human polyomavirus 9
EBV: Epstein–Barr virus
HHV8: Human herpesvirus 8
HPV: Human papillomavirus
IE: Immediate early
gB: Glycoprotein B
FFPE: Formalin-fixed paraffin-embedded
BKV: BK virus.
